# Inhibitory Effect of Whey Protein‐Derived Peptide Leu‐Asp‐Gln‐Trp on Xanthine Oxidase

**DOI:** 10.1002/fsn3.70171

**Published:** 2025-04-17

**Authors:** Kazuya Toda, Masaki Kurimoto, Yuma Hirose, Akio Yamada, Naoki Yuda, Miyuki Tanaka

**Affiliations:** ^1^ Innovative Research Institute Morinaga Milk Industry Co., Ltd. Kanagawa Japan

**Keywords:** Ala‐Leu‐Pro‐Met, Asp‐Gln‐Trp, hyperuricemia, Leu‐Asp‐Gln‐Trp, whey protein‐derived peptide, xanthine oxidase

## Abstract

Hyperuricemia is associated with various diseases, and xanthine oxidase (XO) is the rate‐limiting enzyme in uric acid (UA) production. A previous study reported that Leu‐Asp‐Gln‐Trp (LDQW) in whey protein hydrolysate (WPH) suppressed lipid droplet accumulation in differentiated 3T3‐L1 adipocyte‐like cells. However, our understanding of LDQW remains limited, further, its efficacy against hyperuricemia has not been elucidated. This study evaluated the XO inhibitory activity of LDQW, one of the bioactive peptides in WPH. In this study, UA produced by the reaction between XO and xanthine was determined using two methods: monitoring the absorbance at 290 nm using absorptiometry and detection using liquid chromatography with tandem mass spectrometry (LC–MS/MS) analysis. Allopurinol was used as the positive control, whereas tryptophan and Ala‐Leu‐Pro‐Met (ALPM) were used for comparison. Both absorptiometry and LC–MS/MS analyses demonstrated that LDQW significantly inhibited XO activity in a concentration‐dependent manner. The LC–MS/MS analysis results indicated that LDQW, tryptophan, and ALPM inhibition ratios at 20 mM were 58.0% ± 2.8%, 4.4% ± 3.7%, and 45.0% ± 1.0%, respectively. Moreover, it was suggested that Asp‐Gln‐Trp, a potential digestive peptide predicted by the enzymatic digestion of LDQW *in silico*, also possessed XO inhibitory activity comparable to that of LDQW in LC–MS/MS analysis. These findings suggest that LDQW is a promising bioactive peptide with potential ameliorative effects against hyperuricemia, similar to those of other XO inhibitory peptides.

## Graphical abstract

The inhibition of xanthine oxidase (XO), a key enzyme involved in uric acid production, is an attractive target for ameliorating hyperuricemia. This study demonstrated the inhibitory effects on XO activity of Leu‐Asp‐Gln‐Trp (LDQW), a bioactive peptide in whey protein hydrolysate. Moreover, DQW, its digestive peptide, showed XO inhibitory activity comparable to that of LDQW. These findings suggest that LDQW is a promising bioactive peptide with potential preventive and ameliorative effects against hyperuricemia.

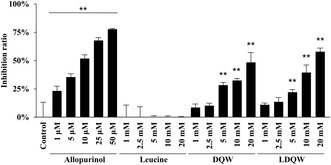

## 1 Introduction

Bioactive peptides can be obtained from various sources, including dairy products, eggs, marine sources, and plants. They have been reported as fragments of proteins comprising 2–20 amino acid residues that demonstrate beneficial biological effects such as angiotensin‐converting enzyme (ACE) inhibitory activity, antioxidant activity, antimicrobial activity, and dipeptidyl peptidase‐IV inhibitory activity (Akbarian et al. 2022; Du et al., 2022). Although the study of bioactive peptides has been accelerating due to the rise of molecular dynamics simulations, many potential bioactive peptides remain unexplored (Du and Li 2022; Vidal‐Limon et al. 2022). Furthermore, existing bioactive peptides may possess additional functions beyond their known ones, necessitating further research on these peptides.

Numerous studies have suggested that hyperuricemia is associated with gout, obesity, hypertension, diabetes, and renal and cardiovascular diseases (King et al. 2018; Yadav et al. 2013). Xanthine oxidase (XO) is a key enzyme involved in the oxidation of the purines hypoxanthine and xanthine to form uric acid (UA) and is a well‐recognized target for alleviating hyperuricemia (Yu, Du, et al. 2024). Some reports have suggested that the risk of hyperuricemia may be reduced by milk and/or dairy product consumption (Dalbeth et al. 2010; Ghadirian et al. 1995; Mena‐Sánchez et al. 2020). Although the mechanism has not been fully elucidated, milk proteins have been reported to decrease serum UA (SUA) levels in healthy subjects (Garrel et al. 1991), suggesting that bioactive peptides derived from milk proteins may be involved in reducing the risk of hyperuricemia. Several peptides derived from milk proteins such as Ala‐Leu‐Pro‐Met (ALPM) and Pro‐Glu‐Trp (PEW) inhibit XO (Qi et al. 2022a, 2022b), and some of these peptides have also demonstrated efficacy against hyperuricemia in vivo (Qi et al. 2022a, 2023).

Several reports have commonly measured absorbance to determine the XO inhibitory activity of peptides (Qi et al. 2022a, 2022b; Umamaheswari et al. 2013; Xu, Liu, et al. 2024). This method measures the absorbance at approximately 290 nm to detect the UA produced by the enzymatic reaction of xanthine with XO and evaluates UA production suppression by adding a peptide. The absorbance method is easy to measure and optimize, and it has the advantage of monitoring the enzyme reaction product, UA, throughout the reaction. Due to these advantages, this method is often used as a representative way to evaluate the inhibitory effects on XO in numerous studies (Mao et al. 2023; Qi et al. 2022a, 2022b; Yu, Du, et al. 2024; Yu, Liu, et al. 2024). However, caution is required when evaluating peptides containing amino acid residues with an absorbance of approximately 290 nm, as tryptophan, for instance, has two peaks at 279.2 and 288.0 nm (Lavrinenko et al. 2020). Their coexistence with UA can significantly interfere with the measured absorbance of UA, potentially leading to erroneous results (He et al. 2019).

Similar issues have been noted when measuring the enzymatic activity of adenosine deaminase using absorptiometry, and a measurement method using liquid chromatography (LC) has been proposed to address these issues (Paul et al. 2005). Measurements using LC can separate reaction products from interfering substances that could affect the measurement values, thus addressing the issues. Some studies have used LC to assess XO inhibitory activity against peptides (He et al. 2019; Mao et al. 2023; Xu, Liu, et al. 2024). In general, LC with tandem mass spectrometry (LC–MS/MS) offers higher sensitivity than high‐performance LC‐ultraviolet detection does because of its ability to detect lower analyte concentrations and reduced susceptibility to matrix effects, suggesting that LC–MS/MS provides excellent sensitivity and specificity and a wide linear range for UA determination (Luo et al. 2013).

Previously, we reported that Leu‐Asp‐Gln‐Trp (LDQW) in whey protein hydrolysate suppressed the lipid droplet accumulation in differentiated 3T3‐L1 adipocyte‐like cells (Hirose et al. 2024). Additionally, LDQW has been suggested to possess several bioactivities, such as remarkable radical‐scavenging capacity (Sadat et al. 2011), inhibitory activity against ACE (Xie et al. 2022), and the potential to adsorb to the surface of bile salts in submicellar or micellar states (Guerin et al. 2016). While LDQW is an attractive whey protein‐derived peptide, our understanding is still limited. For instance, its efficacy against hyperuricemia has not been elucidated.

Therefore, to confirm the potential of LDQW, we investigated its inhibitory effect on XO activity. We evaluated XO inhibitory activity using both conventional absorptiometry and LC–MS/MS and compared the results of both methods.

## 2 Materials and Methods

### 2.1 Materials and Reagents

Peptides with ALPM, LDQW, and Asp‐Gln‐Trp (DQW) sequences were synthesized by BEX Co. Ltd. (Tokyo, Japan), and their purities were over 95%. Tryptophan and leucine were obtained from Kanto Chemical Co. Ltd. (Tokyo, Japan). The evaluation samples such as peptides and amino acids, and allopurinol (FUJIFILM Wako Pure Chemical Corp., Tokyo, Japan) as a positive control for the evaluation method, were suspended in 0.1 M phosphate buffer pH 7.2 (FUJIFILM Wako Pure Chemical Corp., Tokyo, Japan) containing 0.1 M sodium chloride. The samples were obtained for the assay after sonication for 10 min. XO from bovine milk and xanthine were purchased from Sigma‐Aldrich Corp. (MO, USA). XO was suspended in a buffer at a concentration of 100 U/L. The xanthine powder was weighed, 0.5 M sodium hydroxide solution, purchased from Kanto Chemical Co. Ltd. (Tokyo, Japan), was added to a concentration of 16.8 mM, and the mixture was sonicated for 10 min to dissolve transparently. The xanthine solution was prepared by diluting it with buffer at a ratio of 1:10.

### 2.2 Enzymatic Reaction and Monitoring of Absorbance Using a Microplate Reader

**FIGURE 1:**
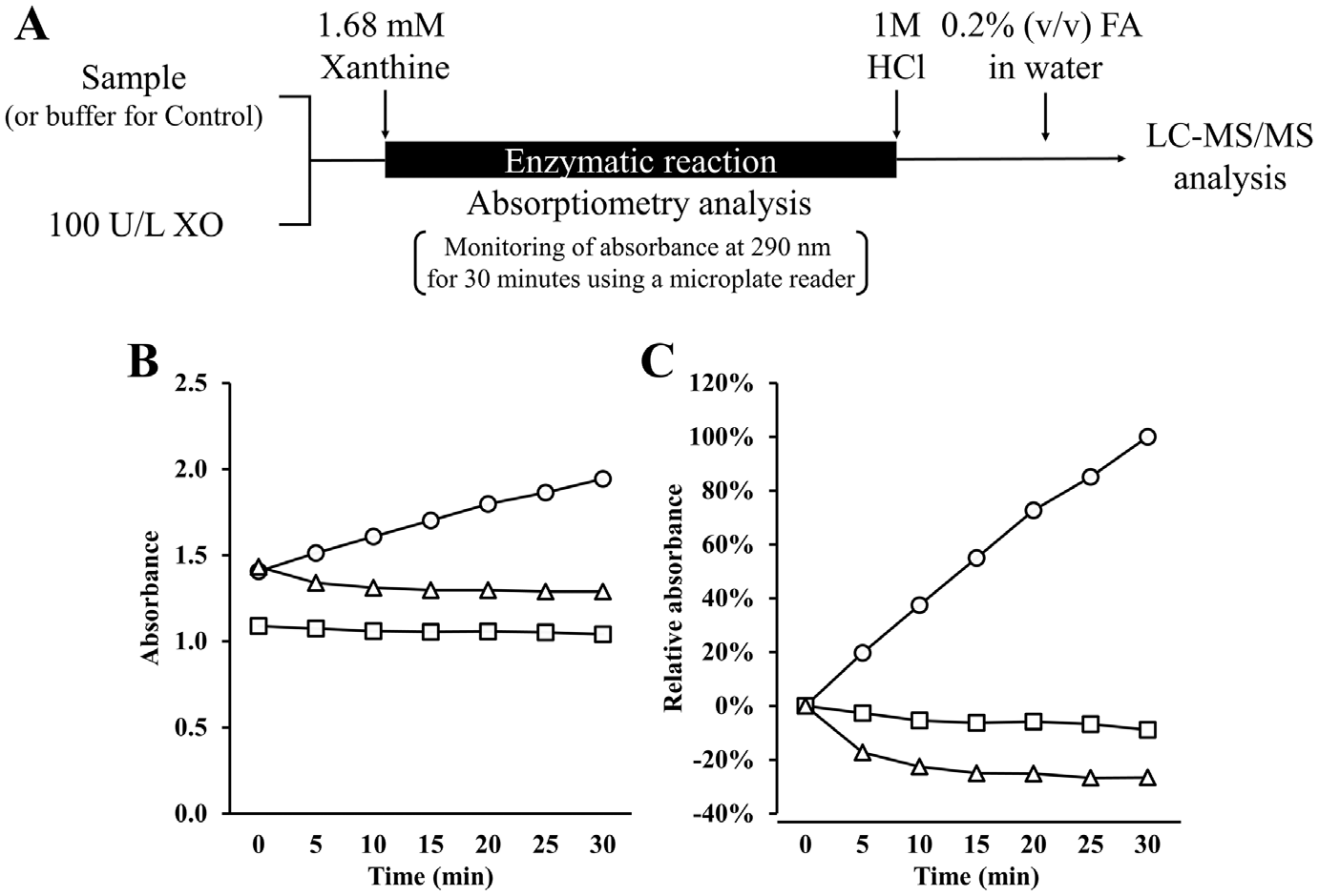
Overview of the evaluation of xanthine oxidase (XO) inhibitory activity. (A) Overview of the evaluation for XO inhibitory activity in this study. Details are described in the Materials and Methods section. To verify the enzymatic reaction, monitoring (B) measured and (C) relative absorbance at 290 nm using a microplate reader. (C) The change in absorbance from 0 to 30 min of the control reacted with XO and xanthine was defined as 100%, with the relative absorbance expressed as a percentage. The symbols represented monitoring of only XO (□), only xanthine (△), and both XO and xanthine (○), respectively.

The enzymatic reaction was performed as described previously (Qi et al. 2022a, 2022b; Xu, Gong, et al. 2024) with slight modifications (Figure 1A). Briefly, the samples (e.g., LDQW) or allopurinol were mixed with the XO solution (final concentration: 25 U/L). A mixture added to the buffer instead of the sample was analyzed as a control. The absorbance at 290 nm was monitored every 5 min for 30 min using a microplate reader SH‐9000 Lab (Corona Electric Co. Ltd., Ibaraki, Japan) as soon as the xanthine solution was added to the mixture (final concentration: 0.42 mM). The sample, XO solution, and xanthine solution were reacted in a 2:1:1 ratio. The assay was performed in triplicate. The XO inhibitory activity was calculated using the following equation:

$$\mathrm{XO}\;\text{inhibitory activity}\;\left(\%\right)=\left[1-\frac{{\mathrm{AbS}}_{30\min}-{\mathrm{AbS}}_{0\min}}{{\mathrm{AbC}}_{30\min}-{\mathrm{AbC}}_{0\min}}\right]\times 100$$

${\mathrm{AbS}}_{30\min}$: Absorbance of the sample at 30 min.

${\mathrm{AbS}}_{0\min}$: Absorbance of the sample at 0 min.

${\mathrm{AbC}}_{30\min}$: Absorbance of the control at 30 min.

${\mathrm{AbC}}_{0\min}$: Absorbance of the control at 0 min.

### 2.3 Determination of XO Inhibitory Activity Using LC–MS/MS

To halt the enzymatic reaction, an equal volume of 1 M HCl, purchased from Kanto Chemical Co. Ltd. (Tokyo, Japan), was promptly added to the mixture after the absorbance was monitored for 30 min (Figure 1A). The mixture was then diluted 50‐fold with water containing 0.2% (v/v) formic acid (FA), which was purchased from Kanto Chemical Co. Ltd. (Tokyo, Japan), to prepare analytical samples for LC–MS/MS. UA in the analytical samples was measured using a Vanquish Ultra‐High‐Performance LC system coupled with a Vanquish variable wavelength detector VF‐D40 and a Q Exactive Focus (Thermo Fisher Scientific Inc., MA, USA). UA in the analytical samples was separated using an XBridge Peptide BEH C18 Column (2.1 mm *i.d*. × 250 mm, 3.5 μm, Waters Inc., MA, USA).

UA separation was achieved using 0.1% (v/v) FA in water (solvent A) and 0.1% FA in methanol (solvent B, Kanto Chemical Co. Ltd., Tokyo, Japan) with the following gradient over 22 min: 2% B for 5 min, increased from 2% to 10% B over 3 min, from 10% to 80% B over 2 min, held at 80% B for 2 min, decreased from 80% to 2% B over 0.1 min and held at 2% B for 10 min. The column temperature and flow rate were maintained at 40°C and 0.2 mL/min, respectively. During separation, the absorbance was monitored at 290 nm.

MS detection was performed in positive ion mode. The optimal ionization conditions were as follows: capillary temperature at 275°C, auxiliary gas flow rate at 10 Arb, sheath gas flow rate at 45 Arb, spray voltage at 3.5 kV, and collision energy at 30%. The UA in the analytical samples was determined by parallel reaction monitoring (PRM) with the precursor ion/product ion at *m/z* 169.03/70.04. The peak area detected at a retention time of 4.3 min was analyzed using Thermo Scientific Xcalibur. UA was quantified using the absolute calibration curve method, which was constructed from the peak areas of UA at various concentrations. XO inhibitory activity was calculated as [1 − UAs/UAc] × 100%, where UAs and UAc represent the amount of UA in the samples and control, respectively.

### 2.4 *In Silico* Prediction of LDQW Cleavage Sites by Digestive Enzymes

*In silico* analysis was performed using the Expasy‐Peptide Cutter tool, as described in a previously published procedure available online (https://web.expasy.org/peptide_cutter/) (Wilkins et al. 1999). The cleavage sites of LDQW by chymotrypsin‐low specificity (C‐term to [FYWML], not before P), pepsin (pH 1.3), and trypsin were predicted using this bioinformatics tool to forecast peptide hydrolysis sites (accessed on 24 December 2024).

### 2.5 Statistical Analysis

All experiments were performed in triplicate, with data indicated as mean ± standard deviation (SD). Statistical analyses were performed using the EZR software (ver. 4.2.2) (Kanda 2013). One‐way analysis of variance (ANOVA) was used for parametric analyses, followed by the Tukey–Kramer test. *p* values of < 0.05 were considered statistically significant.

## 3 Results

### 3.1 Determination of XO Inhibitory Activity Using Absorptiometry

**FIGURE 2:**
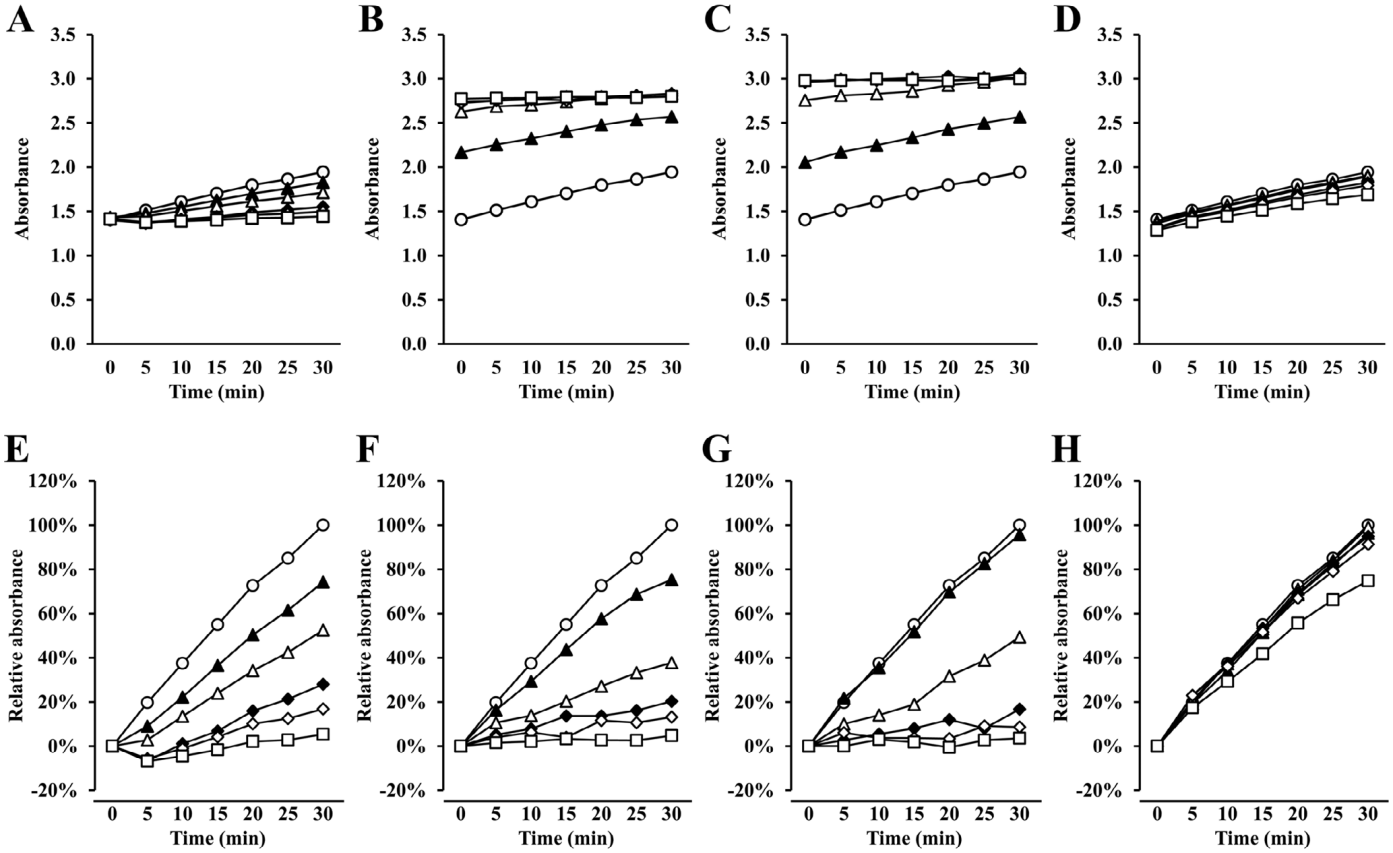
Monitoring measured and relative absorbance at 290 nm using the microplate reader. The figures indicate the means of the measured (A–D) and relative (E–H) absorbance values every 5 min for 30 min (*n* = 3). (E–H) The change in absorbance from 0 to 30 min of the control was defined as 100%, with the relative absorbance expressed as a percentage. The symbols represent the concentration of (A, E) allopurinol, (B, F) tryptophan, (C, G) LDQW, or (D, H) ALPM; ○: Buffer (control); ▲: 1 mM for tryptophan, LDQW, and ALPM (1 μM for allopurinol); △: 2.5 mM (5 μM); ◆: 5 mM (10 μM); ◇: 10 mM (25 μM); □: 20 mM (50 μM). As these were conducted simultaneously, control (○) was common across all figures.

As shown in Figure 1A, XO and xanthine were mixed and allowed to react enzymatically, and the amount of UA produced was measured. In absorptiometry, UA production was monitored by measuring the absorbance at 290 nm. UA production inhibition by adding the sample was determined by comparison with that of the control without the sample. The control, which involved the reaction between XO and xanthine, showed a time‐dependent increase in absorbance. In contrast, the absorbance did not increase when either XO or xanthine alone was used (Figure 1B,C). Furthermore, allopurinol, the positive control, suppressed the increase in absorbance in a concentration‐dependent manner (Figure 2A,E), indicating that the enzymatic reaction functioned properly under the method used in this study.

### 3.2 Inhibitory Effects of LDQW on XO Activity by Absorptiometry

LDQW and tryptophan exhibited absorbance at 290 nm before the reaction, which remained constant from start to finish, especially at high concentrations (Figure 2B,C). The calculated relative absorbance of LDQW and tryptophan indicated that the enzymatic reaction by XO was suppressed compared with the control (Figure 2F,G). At 20 mM, ALPM, which did not show absorbance at 290 nm, suppressed this increase compared with the control (Figure 2D,H).

**FIGURE 3:**
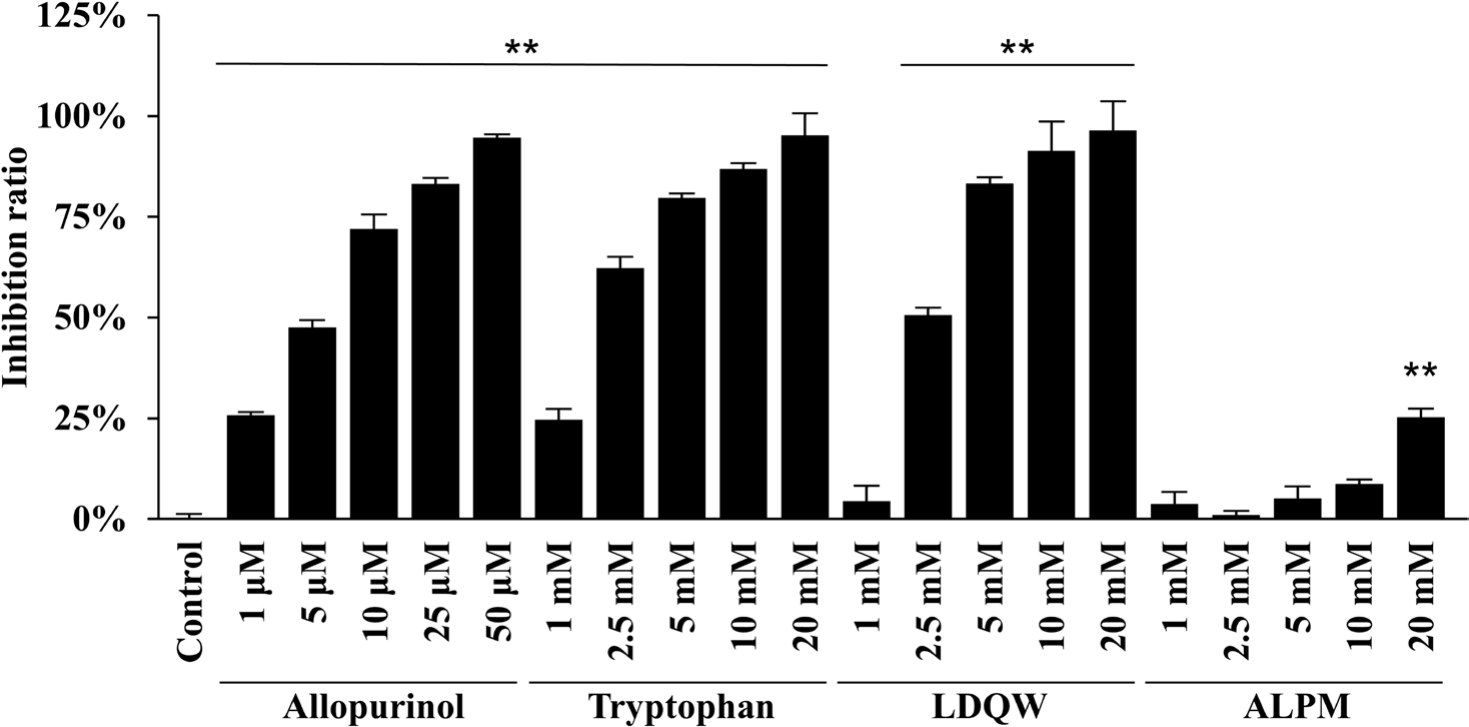
Inhibitory effects of the peptides on xanthine oxidase (XO) activity measured by absorptiometry. The XO inhibition rate, compared with the control, was calculated as described in the Materials and Methods section. Data are presented as mean ± SD (*n* = 3). Statistical analysis was conducted using one‐way ANOVA, followed by the Tukey–Kramer test; **p < 0.01 (vs. control).

XO inhibitory activity was calculated according to the difference in absorbance at the start and end of the reaction by comparing the reaction with and without the sample (Figure 3). Consequently, significant inhibition was observed for allopurinol, tryptophan, LDQW, and ALPM at concentrations of ≥ 1 μM, ≥ 1 mM, ≥ 2.5 mM, and 20 mM, respectively. The inhibitory activities of allopurinol, tryptophan, and LDQW increased with rising concentrations in a concentration‐dependent manner. In the results using a microplate reader, the XO inhibitory activities of tryptophan, LDQW, and ALPM at 20 mM were 95.2% ± 5.3%, 96.4% ± 7.0%, and 25.2% ± 1.9%, respectively.

### 3.3 Detection and Quantification of UA Using LC–MS/MS

**FIGURE 4:**
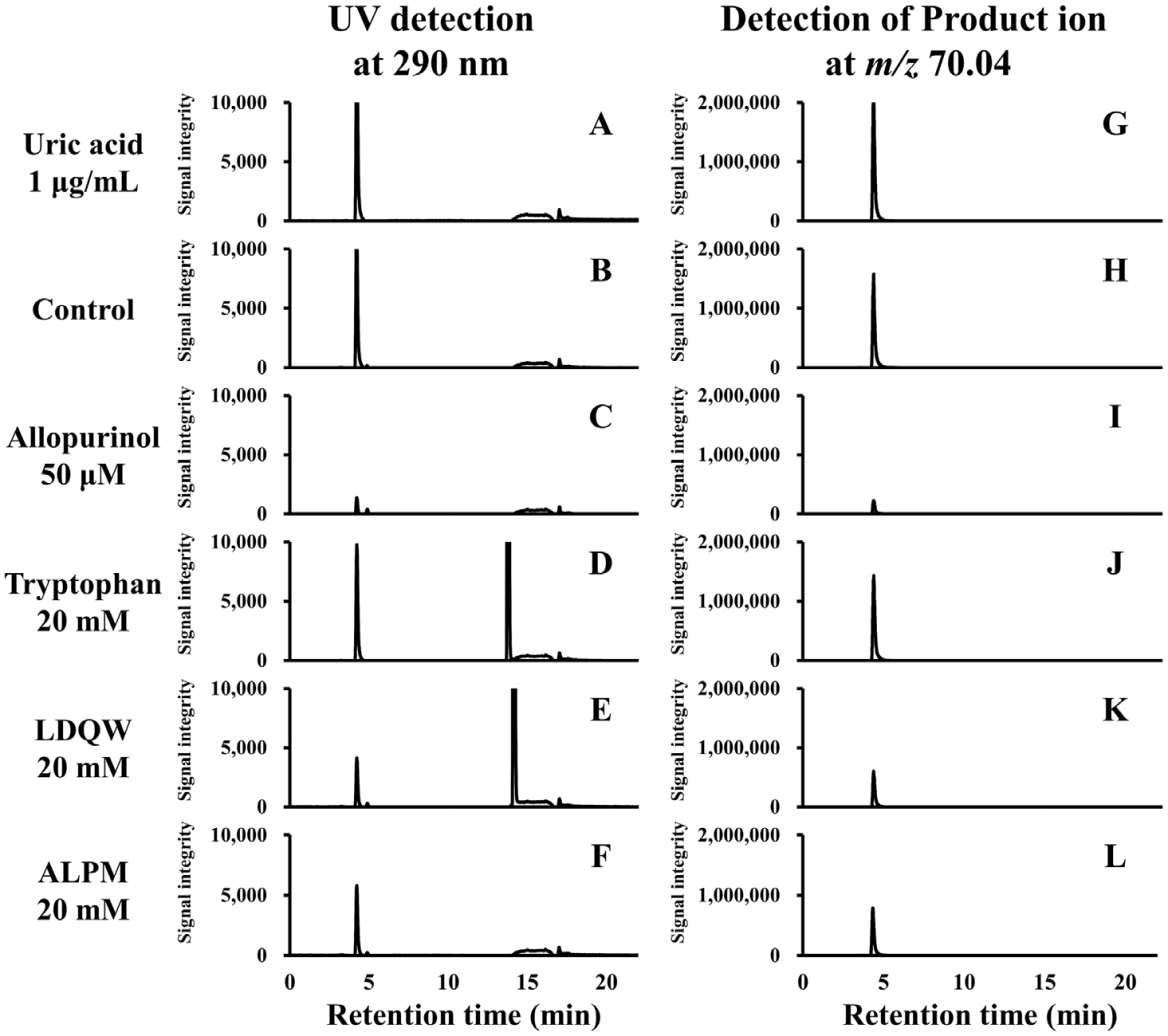
Representative chromatograms at 290 nm and *m/z* 70.04 in LC–MS/MS. Representative chromatograms are shown for analysis at (A–F) 290 nm and (G–L) the production at *m/z* 70.04. These chromatograms indicated (A, G) uric acid (UA) 1 μg/mL as a standard, (B, H) control, (C, I) allopurinol at 50 μM, (D, J) tryptophan at 20 mM, (E, K) LDQW at 20 mM, and (F, L) ALPM at 20 mM, respectively. The UA peak was detected at a retention time of 4.3 min. Tryptophan and LDQW were detected at retention times of 13.7 and 14.1 min, respectively, by the absorbance at 290 nm.

UA detection was performed using LC–MS/MS as detailed in the Materials and Methods section. The analysis revealed a peak corresponding to UA with a retention time of 4.3 min (Figure 4). The precursor ion for UA was identified at *m/z* 169.03, and the major product ions were detected at *m/z* 70.04 and 96.02 (Figure S1). In PRM mode, UA was quantified by the area of the peak at *m/z* 70.04. Additionally, the absorbance at 290 nm confirmed the presence of peaks corresponding to tryptophan and LDQW, which were clearly separated from the UA peak (Figure 4D,E).

### 3.4 Inhibitory Effects of LDQW on XO Activity by LC–MS/MS Analysis

**FIGURE 5:**
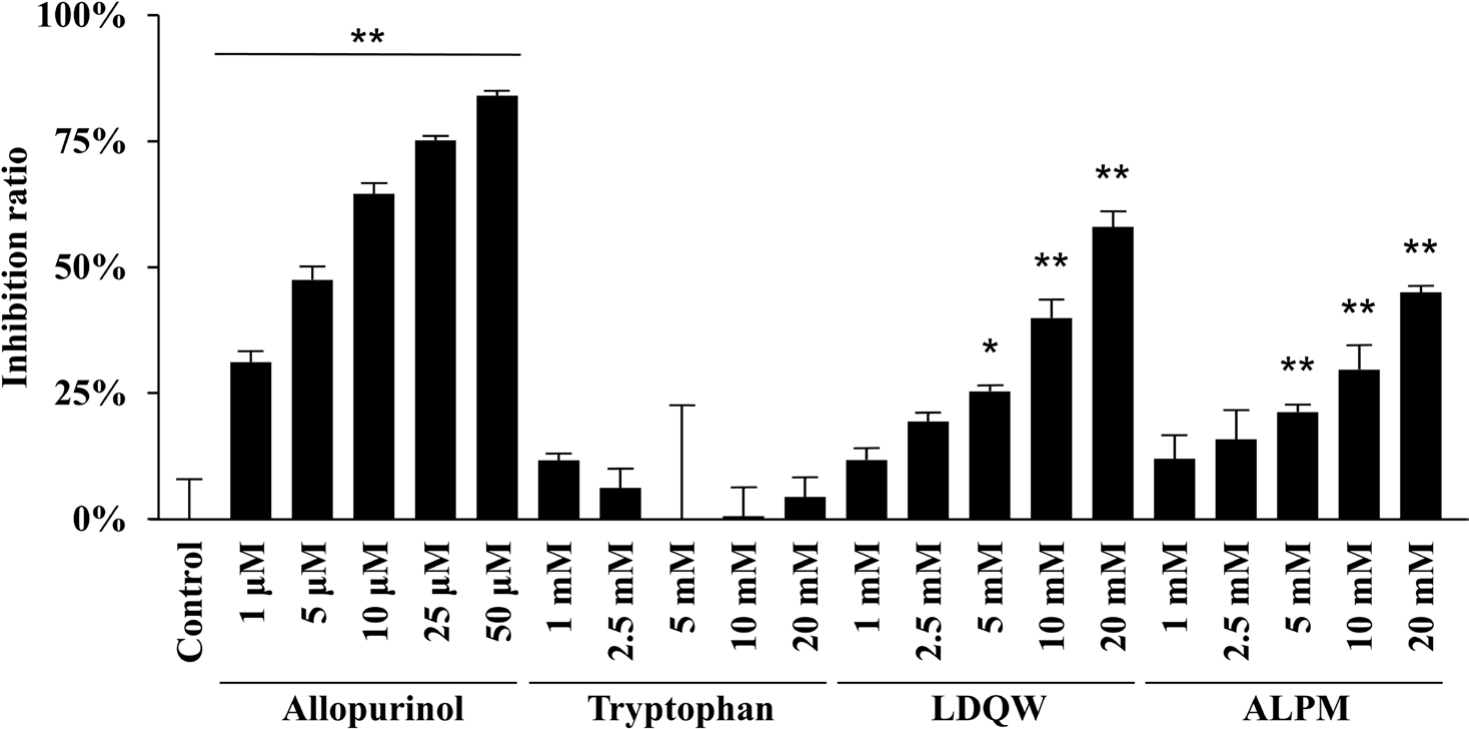
Inhibitory effects of LDQW and ALPM on xanthine oxidase (XO) activity measured by LC–MS/MS analysis. The XO inhibition rate, compared with the control, is presented as mean ± SD (*n* = 3). Statistical analysis was conducted using one‐way ANOVA, followed by the Tukey–Kramer test; *p < 0.05, **p < 0.01 (vs. control).

As shown in Figure 5, 1 μM or higher allopurinol significantly inhibited XO activity in a concentration‐dependent manner. Similar to allopurinol's concentration‐dependent inhibition, LDQW and ALPM also exhibited an increase in the inhibition ratio in a concentration‐dependent manner, with significant inhibition observed at concentrations of 5 mM or higher. The XO inhibitory activities of LDQW and ALPM at 20 mM were 58.0% ± 2.8% and 45.0% ± 1.0%, respectively. Meanwhile, tryptophan did not exhibit significant XO inhibition at any concentration, with an inhibition ratio of 4.4% ± 3.7% at 20 mM.

### 3.5 Inhibitory Effects of DQW, a Fragment Peptide of LDQW, on XO Activity by LC–MS/MS Analysis

**FIGURE 6:**
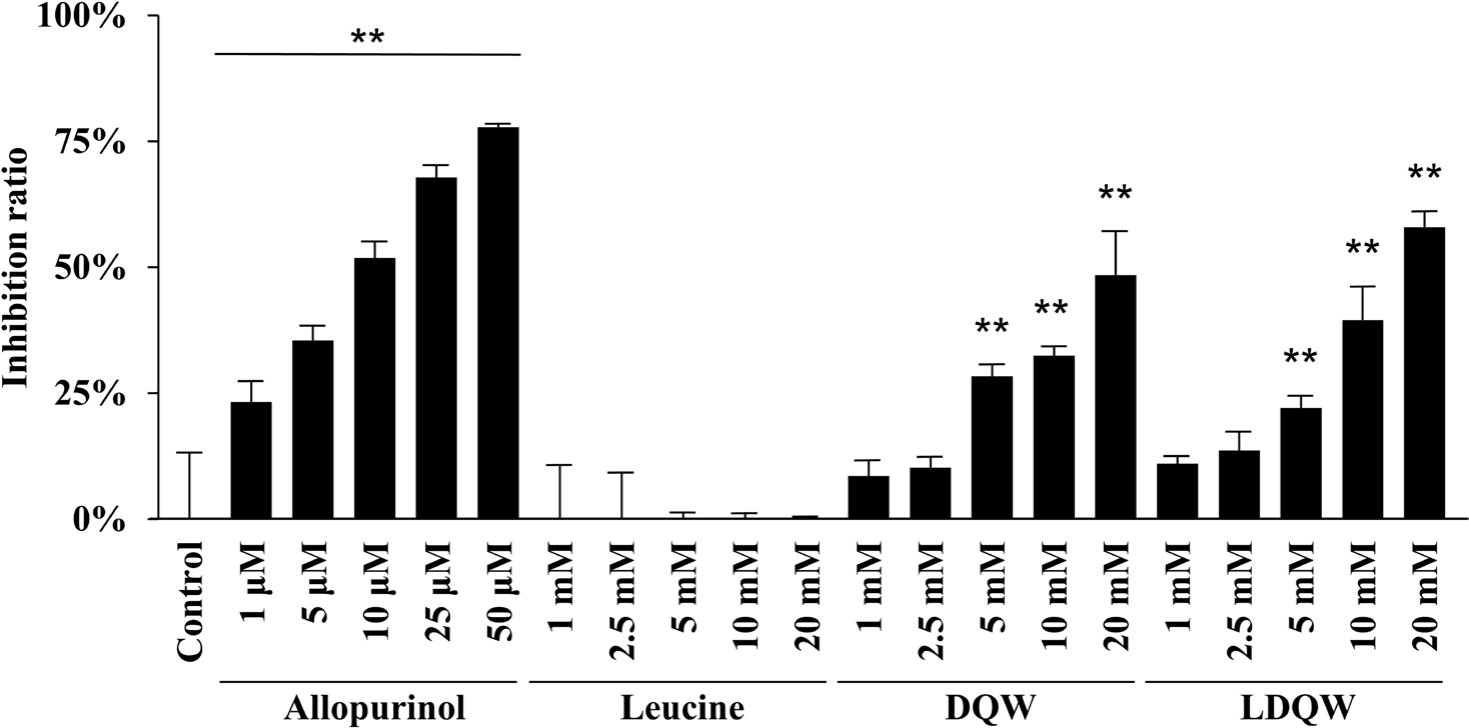
Inhibitory effects of DQW, a fragment peptide of LDQW, on xanthine oxidase (XO) activity measured by LC‐MS/MS analysis. The XO inhibition rate, compared with the control, is presented as mean ± SD (*n* = 3). Statistical analysis was conducted using one‐way ANOVA, followed by the Tukey–Kramer test; **p < 0.01 (vs. control).

Next, we attempted to gain insights into whether the orally ingested LDQW, after being digested by digestive enzymes, also possessed inhibitory activity in its fragment peptides similar to that of LDQW. As LDQW was predicted to be cleaved into leucine and DQW according to the results from Peptide Cutter, we evaluated the XO inhibitory activity of leucine and DQW. The results showed that DQW significantly inhibited XO activity in a concentration‐dependent manner, similar to the inhibition observed with allopurinol and LDQW (Figure 6). The XO inhibitory activities of LDQW and DQW at 20 mM were 57.9% ± 3.2% and 48.4% ± 8.5%, respectively. Meanwhile, no significant XO inhibition was observed for leucine at any concentration.

## 4 Discussion

We aimed to explore the potential of LDQW as a bioactive peptide by measuring its XO inhibitory activity using two evaluation methods: absorptiometry and LC–MS/MS analysis. In both evaluation methods, LDQW significantly inhibited XO activity in a concentration‐dependent manner, and the activity was also observed in this study with allopurinol (Klein et al. 1996) and ALPM (Qi et al. 2022a), which have been reported to have XO inhibitory activity. Additionally, the cleavage sites of LDQW by digestive enzymes were predicted *in silico*, and DQW, its digestive peptide, demonstrated XO inhibitory activity comparable to that of LDQW.

Tryptophan demonstrated a concentration‐dependent XO inhibitory activity in absorptiometry; however, no efficacy was observed in the LC–MS/MS results. As shown in Figure 2, tryptophan exhibited an absorbance at 290 nm and reached a plateau before or immediately after the enzyme reaction. Therefore, it was determined that this caused a contradiction in the tryptophan results between the absorptiometry and LC–MS/MS methods. For example, for ALPM, the inhibition rate at 20 mM measured by absorbance photometry is 25.2% ± 1.9%, which is different from the inhibition rate at 20 mM by the LC–MS/MS method of 45.0% ± 1.0%. Although absorptiometry is a simple and commonly used evaluation method, LC–MS/MS offers high sensitivity and specificity for quantifying UA as a reaction product. Therefore, this difference in results was speculated to be due to the sensitivity and specificity.

There have been numerous reports on bioactive peptides with XO inhibitory activity. Some of them are as follows: IC_50_ of Gly‐Leu: 10.2 mM, Pro‐Met: 23.8 mM, Ala‐Leu: 34.5 mM, Ala‐Met: 40.5 mM, Phe‐His: 25.7 mM, and Tyr‐Asn‐Ile‐Thr‐Gly‐Trp: 9.8 mM (He et al. 2019; Mao et al. 2023; Xu, Liu, et al. 2024). The IC_50_ of LDQW was calculated to be 15.5 mM based on the results obtained using LC–MS/MS, which was considered reasonable in light of previous reports. As the results in LC–MS/MS analysis, LDQW exhibited inhibitory activity comparable to that of ALPM in this study, with inhibition ratios of LDQW and ALPM at 20 mM of 58.0% ± 2.8% and 45.0% ± 1.0%, respectively. Previously, ALPM was reported to affect XO *in silico* through molecular dynamics simulations and molecular docking and to reduce SUA levels and improve renal dysfunction in a rat model of hyperuricemia when orally administered (He et al. 2024; Qi et al. 2022a).

This study has some limitations, such as the fact that this evaluation method does not fully replicate biological processes in vivo. Herein, we discuss the potential of LDQW intake to alleviate hyperuricemia through XO inhibitory activity in vivo, as well as ALPM. SUA is primarily controlled by its production in the liver and elimination by the kidneys (Chen et al. 2016), and there have been several reports associating SUA with liver diseases such as cirrhosis and chronic liver disease (Afzali et al. 2010; Michelis et al. 1974). Additionally, XO has been reported to be highly expressed in the liver (Chen et al. 2016; Sarnesto et al. 1996), specifically localized to periportal hepatocytes, and absent from the perivenous region (Linder et al. 1999). Therefore, considering our findings, if LDQW and/or its digested peptides reach the liver around the portal vein and then affect the XO, it could reduce SUA by suppressing UA production in the liver.

Generally, orally administered peptides have been affected by digestive enzymes in vivo (Amigo and Hernández‐Ledesma 2020; Yu, Liu, et al. 2024). Several absorption pathways have been suggested for food‐derived peptides, including carrier‐mediated transport, endocytosis, and paracellular and passive diffusion (Xu, Gong, et al. 2024). Particularly, some reports suggest that dipeptides and tripeptides are directly absorbed into the body through the peptide transporter such as PepT1 on the intestinal brush border membrane (Adibi 1997; Spanier and Rohm 2018). However, the digestive dynamics of proteins and peptides are complex and have not been fully understood yet. Although further research is required to understand LDQW digestion and absorption, LDQW was predicted to be cleaved into leucine and DQW according to the results from Peptide Cutter. In this study, DQW, as well as LDQW, showed XO inhibitory activity using LC–MS/MS analysis. This implies that oral administration of LDQW may have XO inhibitory activity even if digested by digestive enzymes in vivo, suggesting that LDQW may exhibit XO inhibitory activity irrespective of digestive dynamics.

As study limitations, the mechanism for alleviating hyperuricemia is known to involve not only XO activity inhibition but also other factors, such as UA excretion promotion and UA reabsorption inhibition (Cicero et al. 2021). PEW and Leu‐Leu‐Trp (LLW), known for their XO inhibitory activities, have been reported to reduce the expression of genes related to UA synthesis in the liver and enhance gene expression for UA excretion in the kidneys in a rat model of hyperuricemia (Qi et al. 2023, 2024). Therefore, it has been suggested that PEW and LLW alleviate hyperuricemia through multiple mechanisms. LDQW such as PEW and LLW may improve hyperuricemia through multiple mechanisms in vivo. Therefore, further studies are necessary to investigate the efficacy of LDQW on these mechanisms.

Further studies are needed to validate the hypothesis that LDQW has preventive and ameliorative effects on hyperuricemia. For example, to robustly confirm the inhibitory effects of LDQW on XO, it is essential to demonstrate the potential interaction of LDQW with XO using *in silico* analyses, such as molecular dynamics simulations and molecular docking. Furthermore, it is essential to investigate whether LDQW supplementation can ameliorate SUA levels in vivo and/or in clinical studies.

## 5 Conclusions

In this study, we evaluated the inhibitory effects of LDQW on XO activity using absorptiometry and LC–MS/MS analysis. The results demonstrated that LDQW significantly inhibited XO activity in a concentration‐dependent manner. Although the accumulated evidence is insufficient, these findings suggest that LDQW is a promising bioactive peptide with potential preventive and ameliorative effects against hyperuricemia. Further studies, such as in vivo and/or clinical studies, are required to verify the hypothesis.

## Author Contributions

**Kazuya Toda:** conceptualization (lead), data curation (lead), investigation (lead), methodology (equal), visualization (lead), writing – original draft (lead). **Masaki Kurimoto:** conceptualization (equal), data curation (supporting), methodology (equal), writing – review and editing (equal). **Yuma Hirose:** methodology (equal), writing – review and editing (supporting). **Akio Yamada:** methodology (equal), writing – review and editing (supporting). **Naoki Yuda:** conceptualization (equal), data curation (supporting), project administration (lead), supervision (equal), writing – review and editing (lead). **Miyuki Tanaka:** conceptualization (supporting), funding acquisition (lead), project administration (supporting), supervision (equal), writing – review and editing (supporting).

## Conflicts of Interest

All authors are employed by the Morinaga Milk Industry Co. Ltd. There are no other conflicts of interest.

## Supporting information


Figure S1.

## Data Availability

The data collected and analyzed in this study are available from the corresponding author upon request.
