# Supplementary material for: Inhibitory Effect of Whey Protein‐Derived Peptide Leu‐Asp‐Gln‐Trp on Xanthine Oxidase
Source: Food Sci Nutr. 2025 Apr 17;13(4):e70171. doi: 10.1002/fsn3.70171 (PMC12006031; doi:10.1002/fsn3.70171)
Supplement: Supplementary file 1 — Figure S1. [file FSN3-13-e70171-s001.pdf]

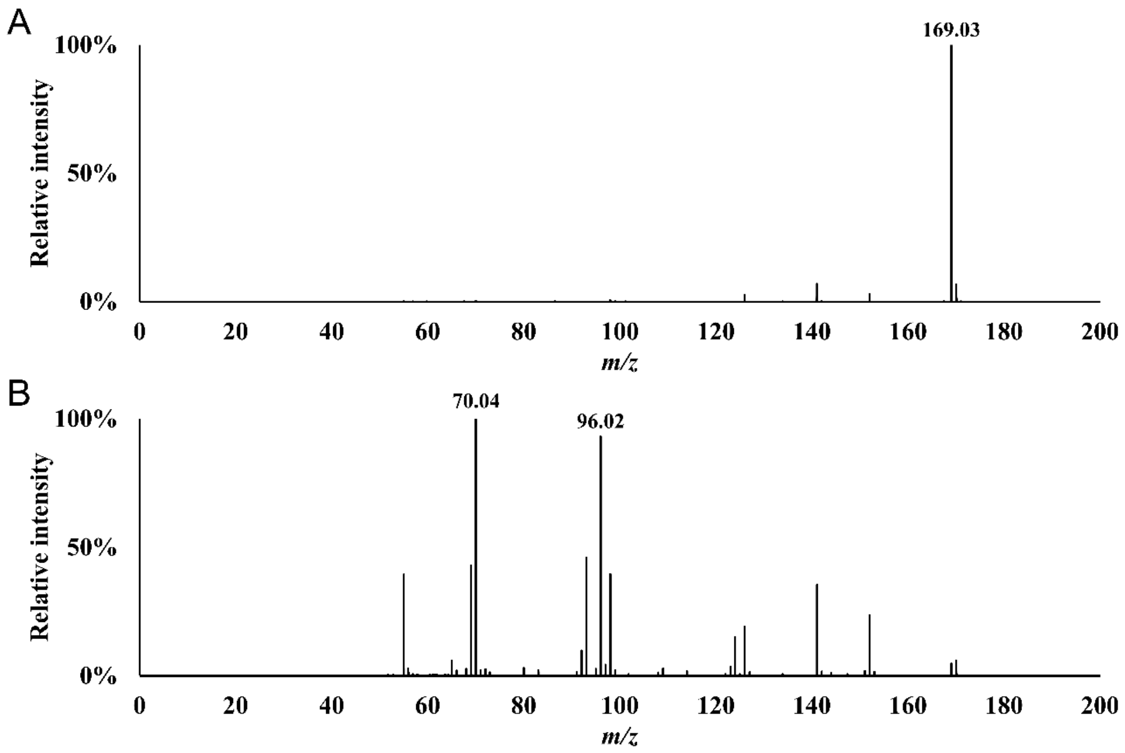

Figure S1. Precursor and product ions in the analysis of uric acid (UA) using LC-MS/MS

Selection of precursor ions (A) and product ions (B) to detect UA using the parallel reaction monitoring. (A) MS spectrum of the precursor ions from UA. (B) Product-ion tandem MS spectrum of the precursor ions at *m*/*z* 169.03.
